# Reviewing fair subject selection considerations for the unique case of post sequelae COVID-19 translational studies

**DOI:** 10.1017/cts.2022.425

**Published:** 2022-07-07

**Authors:** E.M. Smith, E.E. Anderson, R. Deer, J. Prochaska, K. Bohn, S. Croisant

**Affiliations:** 1 School of Public and Population Health, University of Texas Medical Branch, Galveston, TX, USA; 2 Institute for Translational Sciences, University of Texas Medical Branch, Galveston, TX, USA; 3 Loyola University Chicago Stritch School of Medicine, Maywood, IL, USA; 4 School of Health Professions, University of Texas Medical Branch, Galveston, TX, USA; 5 this researcher has recently left UTMB and joined Novartis

**Keywords:** Post sequelae COVID-19, research ethics, fair inclusion, fair subject selection, diversity, representation in research

## Abstract

Fair inclusion of research subjects is necessary to ensure that post-acute sequelae COVID-19 (PASC) research results benefit all members of society. Scientists should conduct research on a broad sample of individuals who represent clinically relevant factors influencing a disease. Without demographic diversity and sociological and environmental variability, research outputs are less likely to apply to different populations and would thus increase health disparities. The goal of this narrative literature review and ethical analysis is to apply fair selection criteria to PASC research studies. We briefly highlight the importance of fair subject selection in translational research and then identify features of PASC, as well as PASC research, that hinder fair inclusion of research participants. We will demonstrate that determining an adequate and representative sample is not simply a matter of ensuring greater diversity; rather, fairness requires a broader evaluation of risks, burdens, and benefits specific to underrepresented populations. We provide recommendations to ensure fair subject selection in PASC research and promote translation toward positive health outcomes for all individuals, including the most vulnerable.

## Introduction

Given the significant disparities in mortality and morbidity linked to COVID-19, scholars have repeatedly called for increased inclusion in biomedical health research of underrepresented populations, notably pregnant women, older populations, incarcerated individuals, individuals with comorbidities, and ethnic and racial minorities [1–4]. Currently, COVID-19 treatment and vaccine trials lack diversity, and inclusion of Black and Latinx participants is particularly limited [2]. Without ongoing systemic corrective action, limited inclusion of underrepresented groups will likely also be the case in research on “long-haulers,” those chronically afflicted with long-term effects related to COVID-19, also referred to as post-acute sequelae of SARS CoV-2 (PASC). When translation of knowledge excludes or fails to benefit all populations, health disparities increase [5,6].

Although it may be tempting to focus on rapidly increasing and diversifying recruitment efforts for PASC research, ethical fair subject selection must include and balance broader principles of fair inclusion, fair burden sharing, and fair opportunity [7]. Fair inclusion requires representation of participants with diverse characteristics relevant to the disease and intervention. Fair burden sharing requires that researchers select participants most able to shoulder risks and burdens from research. Fair opportunity requires reasonable efforts to enhance opportunities to participate and not unfairly discriminate or exclude people from research participation.

Although these concepts of fair subject selection may seem intuitive, their application is fraught with conflicting ethical imperatives [7]. For example, ensuring fair inclusion by including subjects who are clinically relevant to research may conflict with fair burden sharing. This is certainly the case in COVID-19 and PASC research. Disparities in risk, exposure, morbidity, and mortality have been uniformly higher among vulnerable populations such as communities of color and those living in poverty [6,8–14]. Patients – especially underrepresented minorities – infected with COVID-19 and then afflicted by PASC and its extended burden of illness are not only affected by these existing inequalities, but may further be negatively impacted by poor health, work effectiveness, family dynamics, and socioeconomic status, for example, in cases of lost employment. While underrepresented populations have already been burdened by the pandemic, it remains important to include them in research for reasons of clinical relevance and generalizability. To illustrate another example of conflicting ethical imperatives, ideally, researchers would also provide fair opportunity to those who wish to participate. However, including too many people of similar clinical relevance is not appropriate for a representative sample. As such, we may be turning away willing participants that hope for the opportunity to participate in research.

In this paper, we will review concepts related to fair subject selection in the case of PASC research. To do so, we will start by briefly reviewing the ethics of fair subject selection and map out the regulatory landscape that impacts the translational research system. We will then look at features of PASC and current PASC research that threaten fair subject selection and may lead to biased scientific knowledge and limited translation of research results in already marginalized populations. We look at the ethical implications of PASC research and develop recommendations to promote broader inclusion and, ultimately, more trustworthy PASC research. This will lead us to consider important strengths and limitations of fair subject selection principles and identify the need to develop a more systemic inclusion strategy not only for PASC research but also for translational research more broadly. Although PASC research is a specific case study with unique challenges, it illuminates issues of systemic importance that may also be present in other types of translational research. Currently in translational science, efforts to increase diversity of research participants may ignore the full range of considerations relevant to systemic fair subject selection and thus may exacerbate issues of representation, burden, and applicability of research findings.

## Background: Reviewing the Ethics and Policy of Fair Subject Selection in Research and Translational Science

In response to the scandalous abuse and exploitation of vulnerable populations, research ethics frameworks of the 1970s – including the Belmont Report in 1979 and subsequently, in 1981, the Department of Health and Human Services regulations for the protection of human subjects (45 CFR part 46) – offered guidance focused on protecting participants in positions of vulnerability from potential harms associated with research. The concept of “vulnerable population” used in the Common Rule subparts B, C, and D refers to minors, fetuses, and prisoners, with additional mention of “subjects that are likely to be vulnerable to coercion or undue influence, such as (…) individuals with impaired decision making capacity, or economically or educationally disadvantaged persons (…)” [15]. Over time, a more expansive notion of “special populations” has evolved in translational research which encompasses not only the vulnerable populations mentioned in federal regulations but also elderly adults (often with comorbidities), historically under-representation ethnic or racial groups and people living in rural areas, among others [16]. Different terms such as vulnerable, special, underrepresented, or disenfranchised all have slightly different definitions and may be linked to different scholarly and public discussions and debates. However, data suggest that those who are not white, not male, not middle aged, not able bodied, with multiple comorbidities, and without access to medical care remain often underrepresented in clinical and translational research and thus have poorer health outcomes on a population level [17–20]. This is unjust.

Although included in foundational documents such as the Belmont Report, justice remains the least well-established conceptual notion in research ethics compared with the principles of beneficence and respect for persons. London argues that it is the “last virtue of research ethics” [21]. More specifically, justice remains poorly operationalized, resulting in less applicable practical scholarship when compared to other principles (such as autonomy, around which there is an enormous scholarship on the application of informed consent in research) [22,23]. Justice has historically been interpreted as “fair subject selection,” which may include 1) fair burden sharing, 2) fair opportunity, 3) fair inclusion, and 4) fair distribution of third-party risks. Mackay and Saylor include all four subprinciples in their framework for fair subject selection while acknowledging the challenges inherent in conflicting imperatives [7]. Here, in an attempt to define more clearly who should be included in PASC research and how inclusion should be approached, we will apply Mackay and Saylor’s framework to PASC as it is both recent and open to a range of interpretations.

The principle of “fair burden sharing” is the facet of fair subject selection that aims to protect populations from exploitation. Although it is often necessary to impose certain risks and burdens on human subjects including vulnerable, special, or underrepresented populations for the possibility of benefits, such burdens should not be unfairly distributed out of mere convenience to those least able to bear such burdens. Using burdened individuals is considered unfair especially if the research is intended to only benefit other populations, as in the case of AZT studies for the treatment of HIV-AIDS in South-Eastern Asian and African countries including populations that would never have access to the drug once studies would be concluded [24]. Unfortunately, the narrow focus on the protection of “vulnerable populations” became a form of regulatory paternalism wherein populations identified as vulnerable were often excluded from being research subjects [25]. A softer form of paternalism may be justified, for example, limiting inclusion of people who would face an extremely high level of risk [7] or capping acceptable net risks of a research project [26]. However, paternalism should not result in lack of opportunity, as this may result in underrepresentation of certain group, inequitable distribution of benefits of research, and ultimately increased health disparities.

Debates regarding opportunity to participate in research were highlighted during the 1980s HIV/AIDS patient movements [27]. Although the principle of justice as defined in the Belmont Report included fair distribution of burden and benefits, in practice the limiting of risks of harm seems to have taken priority in the days after the Common Rule first went into effect. In the late 1980s and 1990s, patient groups put significant pressures on the US Federal Drug Administration (FDA) to provide more access and opportunity to receive experimental HIV drugs [28]. Mastroianni and Kahn describe a shift from “from justice as protection to justice as access” during this time [25], thus elevating the principle of fair opportunity to participate in a study with the obligation to minimize risk of harm and burden. Although fairly distributing benefit may *a priori* seem less important than distributing harms, the popularity of debates regarding access to experimental medication including for compassionate and expanded use, as well as the “right to try” highlight the ongoing desire for access to research – and the potential for benefit – in the United States [29,30].

In the 1990s and 2000s, federal research policies further broadened subject inclusion to recognize participants as autonomous agents with the freedom to make decisions about participation in risky activities such as research. For example, the 1993 US National Institutes of Health Revitalization Act emphasized the inclusion of women and minorities in clinical research [31]. Many prior clinical trials in the 1970s were conducted based on the “standard model” of the healthy white man with a mean age of 35 years old [32]. It might be argued that such a homogenous sample was most likely to provide positive outcomes publishable in scientific publications; however, this work is not translatable to diverse populations. The fair inclusion principle demands that participants exhibiting the range of clinically relevant factors be included, and methodologically sound subgroup analysis be conducted, to result in scientific findings that apply to the diverse groups in a population. Subgroup analysis is ideally planned in the protocol to ensure adequate statistical design and reporting of the data regarding relevant groups. Methodological and statistical literature does highlight the dishonesty of certain types of *ad hoc* subgroup analysis which aim to find *any* statistically significant results that will facilitate publication; this is similar to issues with p-hacking where researchers try multiple different statistical tests to highlight statistically significant results which may well be false positives [33]. However, if a researcher finds unexpected results that highlight substantial differences between groups, they should be reported as a transparent post hoc finding, which can lead to new hypothesis that should be replicated in future studies [34].

Preclinical research on cells or animals used in translational modes of inquiry has also come under scrutiny more recently. Although it was long thought that including female animals may result in confounding results because of the estrous cycle, this was shown to be untrue [35,36]. Studies on sexual inclusivity in publications from 1980 to 2016 throughout the translational pathway suggest that while there has been increased inclusion in public health and clinical research, it is still lagging in biomedical research [19]. To address this, in 2016, the NIH enacted the Sex as a Biological Variable (SABV) Policy that requires the use of both male and female organisms in preclinical research as well as human research [37].

Despite positive policy shifts in the 1990s and 2000s, studies on minority inclusion and minority health show ongoing disparities in inclusion rates. Although there exists striking racial/ethnic disparities in cancer incidence, a study conducted in 2014 demonstrated that less than 2% of the National Cancer Institute’s clinical trials focus on any racial/minority population as their primary emphasis [38,39]. Similar racial/minority population underrepresentation is present in research of cardiovascular disease, diabetes, and respiratory illnesses [40]. Significant effort will be required to ensure that this does not continue in PASC research; otherwise, it is certainly likely.

Within the research ethics scholarship there has been an increase in attention to *fair distribution of third-party risks*. In other words, research could impact nonparticipants and therefore the distributions of such burdens should be fair and given to those most able to bear them. For example, if there is a finding suggesting that a particular racial group was much more likely to have PASC and thus likely to have continuing issues with daily functioning, this may negatively impact their work prospects if their diagnosis is known by their employers. The most cited case of third-party risks is the studies with the Havasupai tribe [41,42]. In this community-based research, the community provided samples to study genetic markers related to diabetes. They later found out that their samples were used to study inbreeding, alcoholism and the origin and migration patterns. This would impact not only participants but also that whole group.

Inclusion and diversity have been a common theme in translational research given its emphasis to produce research that is relevant, useful, and economically sustainable [43]. Given the desire to reduce the translational gap between clinical relevance and populational relevance, fair inclusion takes on increased moral importance compared to past traditional research that may not have been considered “translational” in nature. A Delphi panel with 63 expert stakeholders in the United states highlights the importance of moving beyond Belmont principles that protect human subjects toward obligations to protect the community at large through cultural appropriateness and community priorities [44].

Research institutions funded by NIH-Clinical and Translational Science Awards (CTSA) have integrated various community engagement practices to increase diversity in dissemination and implementation of research [45,46]. CTSA institutes have also formalized collaborations between large academic medical centers and researchers at minority institutions that may provide much needed reach and cultural competency to help diversify samples while also increasing the training of scientists from underrepresented groups [47]. There exist many reviews of guidelines and considerations of barriers related to recruitment in the translational research space [16]. According to an exploratory mixed-methods survey (N = 279) and interviews (n = 26) at the Clinical and Translational Science Award Institute at the University of Wisconsin, researchers have an abstract belief in the importance of diversity but most do not consider it an important goal in their specific research projects [48,49]. Although community engagement and partnerships are integrated in CTSAs, this is still not viewed as mainstream nor obligatory in clinical research [50], although mechanisms such as the Community Engagement Studio developed by the Vanderbilt-Meharry CTSA are currently being more widely adopted [51]. There is also a heightened awareness mainly in dissemination and implementation of health science that health equity concerns should minimally not be increased by research [52]. However, as we will argue in the next section, the problem of fair subject selection in PASC research gives rise to further health equity concerns.

## Features of PASC and PASC Research

### Lack of Clear Definitions of PASC

Symptoms that persist or appear after the typical convalescence stage of COVID-19 infection have various labels including “Post-Acute COVID-19,” “Long COVID,” “COVID-19 Syndrome,” and “Post-Acute Sequelae of COVID-19” (PASC). The conceptualization of disease determines who is studied. Since the pathophysiology of PASC is not yet understood, PASC is not yet well defined and therefore who is being studied as well as who should be studied is unclear. The emergence of post-sequelae symptoms was not in itself surprising since many other infectious diseases – such as *Borrelia burgdorferi* (Lyme disease) and Epstein-Barr virus may trigger post-infection multisystem symptoms (fatigue, pain, cognitive issues, and mental health issues) [53]. Physicians currently do not know the exact mechanisms underlying PASC symptoms and therefore rely on perceived characteristics of the disease to diagnose PASC, e.g., headache, fatigue, and anosmia. This lack of clear diagnostic criteria makes it hard to know who has PASC and therefore who to include in PASC research.

Research to understand PASC started with surveys-based analysis relying heavily on self-reporting of lingering symptoms from a COVID-19 infection [54]. To better understand the long-term effects of COVID-19, researchers started monitoring COVID-19 patients after discharge from hospital [55]. A review of prospective studies on the natural history of PASC suggests that the majority of studies recruit hospitalized patients; it remains unclear if ICU patients, hospitalized patients, and nonhospitalized patients have different PASC symptoms and needs [56]. Research at the end of 2021 and in 2022 suggests that people who are not hospitalized also have significant PASC symptoms [57–60].

The first 15 published studies on PASC identified 55 different symptoms, while patient-led initiatives have identified over 205 PASC-related features [53]. Studies use different terms for similar symptoms and cover very different timelines ranging from two weeks after onset of symptoms to more than 6 months [61]. Anti-SARS-CoV-2 antibodies are not always present or look identical to post-vaccination antibodies, leaving little evidence to be tested or clear diagnostic measures [62]. Although digital epidemiology methods seek common, long-lasting cytokine signatures that may facilitate diagnostic criteria [63], this novel approach is still at a preliminary stage.

Although specificity and clear definitions of PASC are necessary for research and clinical care, overly limiting diagnostic criteria may be equally problematic. In understanding PASC, we must fully grasp the biological, cultural, or environmental factors that may create symptomatic differences over time – in the present, for the medium, and for the long term. Researchers have suggested that COVID-19 is a “Syndemic” influenced by many social and biological phenomena including noncommunicable diseases, health resource strain, socioeconomic disparities, unequal housing, and racism [64]. Thus, fully understanding the drivers of COVID-19 spread, as well as identifying effective intervention strategies to counter this pandemic, becomes increasingly complex. The challenge remains to identify and define the complexity of PASC while also creating a delimited disease, illness, or syndrome that can be accurately studied.

### Biased Diagnosis

To further complicate matters, currently, there are inherent biases in who gets diagnosed with PASC, independent from symptomatology. Inclusion in a PASC study often requires a PASC diagnosis typically linked to a past COVID-19 diagnosis and a history of long-lasting symptoms. However, many individuals with PASC symptoms never tested positive for COVID-19. This is a significant issue; a patient-led research study published in pre-print in November 2020 found that 47.8% of its 640 participants were not able to get tested and 27.5% tested negative despite COVID-19 symptoms [65]. In some cases, individuals with PASC symptoms may have received false negative results after COVID-19 testing or experienced lack of access to testing given the many barriers such as transportation, being asymptomatic, or having a mild case. At the beginning of the pandemic, COVID-19 testing allocation was based on medical need – people hospitalized or people most at risk of serious illness were most likely to get tested [66]. At that time, PASC was unknown and there was no awareness that not getting testing might exclude patients from future PASC studies. Individuals from non-white racial groups are much less likely to have access to testing [67,68], which would exclude them from many studies outright. Individuals with low health awareness/health literacy will be less likely to recognize their symptoms or seek treatment.

Others with PASC symptoms may willingly choose not to see a doctor to inquire further about new health concerns. Notably, individuals who are underinsured or uninsured will be much less likely to incur the cost of consulting a doctor regarding common symptoms such as headaches or body pain that may or may not be attributable to PASC. Those with lower incomes may not be able to incur the loss of wages associated with unpaid time off to see a doctor or may fear that a diagnosis of persistent illness may result in permanent job loss. Individuals who have lived testimonial injustice in the past may also be likely to forgo discussing certain symptoms. Testimonial injustice occurs when a person’s voice is undervalued or discounted because of bias often linked to social identity [69]. Evidence does indeed suggest that higher standards of evidence are necessary for certain underprivileged groups before they are believed [70]. After being systematically disbelieved, individuals may feel silenced which may in time turn into self-censoring.

### PASC Stigma

A PASC diagnosis carries potential stigma. Many PASC symptoms affecting mental health, fatigue, or mobility have historically been perceived negatively. For example, diseases with similar symptoms such as chronic fatigue disorder and fibromyalgia are often viewed as excuses concocted to willfully shirk obligations, operate at reduced capacity at work and be less “functional” according to the socially normalized ableist view. Those without access to paid leave and those most likely to be paid lower hourly wages may feel compelled to ignore or endure any symptoms related to PASC to avoid being perceived as less productive [64]. This stigma will not only reduce ability to get a proper diagnosis leading to care, it will also lead to the population being excluded in research.

Similarly, those suffering from brain fog and depression may experience the same stigma typically attributed to mental health issues. There is already significant reluctance to seek treatment for mental health issues among many minority populations – this may complicate diagnosis of PASC and serve as an additional barrier to research inclusion [71]. The stigma may be exacerbated in Black and Latinx populations already disproportionately affected by COVID-19 or among individuals of Asian descent who have faced discrimination due to the origins of the initial outbreak of COVID-19 in Wuhan, China [72].

There has been significant resistance by patient groups to compare PASC to chronic diseases or syndromes that are stigmatized [73]. To avoid words such as “syndrome” or “chronic,” patient groups have argued that the term “long COVID” be used deliberately and consistently to avoid stigmatization [74]. Often seen as psychological in nature, postinfectious syndromes such as chronic fatigue syndrome/myalgic encephalomyelitis (CFS/ME) have been highly controversial in research and clinical practice because of lack of clear diagnostic criteria, causal factors/etiology, and treatment options.

Although resistance to link PASC to stigmatized issues is understandable, one could also learn from the research issues regarding such diseases. For example, most initial studies on CFS/ME included predominantly white women who are of mid to high socioeconomic status [75,76]. The public believed that it was a disease that mainly affected that white women leaving other racial and ethnic groups undiagnosed. As is the case with PASC diagnosis, there is also significant testimonial injustice with ethnic minorities or populations leading their symptoms to be undervalued by health care physicians. However, research conducted on a randomized community sample demonstrated that CFS/ME is found consistently amongst women, minority groups (mainly Latinx and African American groups), and persons of lower levels of education and occupational status [77,78]. Although there is an increase in research on ethnic and racial minorities in CFS, there remain significant gaps in the literature [79].

Although subgroup analysis is critical to understanding differences in disease characteristics and responses to treatments, it is not without risks. In the case of PASC research, a finding suggesting that a racial group has lower incidence of PASC or certain PASC symptoms may lead an employer to inappropriately question an employee’s diagnosis of requests for accommodations. This would impact not only research participants but may also stigmatize a whole group; as such this is considered a third-party risk of research.

### PASC Research Lacks Common Measurements

Since PASC is so new, there exists no standard framework to evaluate symptoms or clinical indicators [53]. Although data from studies using different methods can be triangulated, this significant lack of ontological coherence within PASC research creates challenges in knowledge development; notably, this undermines the ability to compare and combine/pool findings from multiple studies [53,61]. Some have suggested various core domain sets (e.g., depression, pain, and QoL) and core outcomes sets (actual scales; EURoQOL scale, depression scale) but further work is necessary to allow for commensurable findings. Without common scales, we cannot measure and understand how, and the extent to which, certain groups differ with regard to symptomology and treatment efficacy [57]. Given the significant inequities linked to PASC, researchers will be required to go beyond inclusion and develop and report sophisticated subgroup analyses. Combining multiple studies could facilitate this but that would require common measurements across studies. Specific methodological applications of subgroup analysis are beyond the scope of this paper and certainly requires more discussion.

In determining scales, a central question is: how and for whom are such scales established in the first place? The identification of scales or measurements will always assign more or less value to specific aspects of an illness. Some aspects may be more relevant or important to certain people or groups than others. For example, a lack of mobility may be significant to person A who runs marathons for personal enjoyment compared to person B who has chosen a more sedentary lifestyle but requires a lesser amount of mobility for their work. The function evaluation in absolute numbers has declined more in person A compared to person B. However, if person B cannot work because of a small limitation in mobility this may be more problematic. In this case, measurement of level of mobility would find A’s situation more dire. Alternately, measurement of the impact on employment or quality of life may find B’s situation more problematic. Person B may feel constrained or limited, but the difference in wellbeing although significant may not justify an expensive trip to the doctors if Person B does not have insurance. And, even if they did go to the doctor thinking they had PASC, participation in a study related to such issues may seem completely unreasonable especially if no benefit was to be sought or obtained. Although it is certainly necessary to find measurements for research that can be used systematically, it is important to be mindful that measurement decisions are value based and may be more aligned with the values of more privilege groups.

### Lack of Demographic Diversity

Most PASC research is conducted in PASC clinics affiliated with academic medical centers, which have been created to provide support and care to individuals with long-term symptoms after COVID-19 infections. These clinics are typically multidisciplinary to address the diverse health issues that affect physical function, cognitive function, and mental health. These clinics were also established with the important research goal to follow patients longitudinally to identify and analyze clinical patterns while also integrating various research efforts [80]. In effect, this integrated research approach reiteratively studies treatment to improve care. Many PASC clinics, including John Hopkin’s PACT clinic and University of California-San Francisco IMPACT clinic, have two referral pathways – one for hospitalized patients and an alternating referral pathway for COVID-19 survivors with persistent pulmonary or rehabilitation needs [80]. As facilities capable of data collection, these clinics have become central to observational research about PASC and for recruitment of PASC patients for intervention studies [80–82]. However, they serve a select population of PASC sufferers, which means that they will be limited as a source of representative samples for research, in terms not only of demographics (as PASC clinics are more likely to serve insured, urban residents) but also in terms of disease characteristics. For example, at the PASC clinic at UTMB, the significant overrepresentation of women with PASC suggests that recruiting men for PASC studies may be difficult and recruiting men from diverse racial groups even more challenging.

### Limited Potential for Direct Benefit

Since potential treatments are limited, early PASC research has been primarily observational [83]. Research engagement – particularly for those who did not believe they were “sick” and who were evaluated and diagnosed for research purposes – may contribute to increased stress and worry. Moreover, individuals from communities historically exploited by researchers with limited access to healthcare may find it unacceptable to be observed and not treated all in the interest of biomedical science and for the eventual benefit to the more privileged individuals with better access to healthcare. Although research ethics will always promote a “favorable risk-benefit ratio” there are many research studies that simply offer no benefit (referred to as no ex-ante net benefit – no forecast of benefit). This is certainly not unique to PASC, nor is it prohibited from a regulatory or legal standpoint. For example, Phase I clinical trials are conducted on healthy individuals to evaluate the dosage tolerance of a medication. While these individuals are not sick and would not obtain any benefit from this type of research, the study is justified based on the broader social benefit likely to be gained [84]. In the USA and elsewhere, limitations have been established respecting the risks of research on populations that are “vulnerable,” but they usually target federally recognized populations (children, neonates, and prisoners). The notion that this may leave other disenfranchised populations at higher risk without known benefit may be seen as morally problematic. We will discuss how this links to burden sharing in the next section.

### Ethical Implications of PASC/PASC Research for Fair Subject Selection

The aforementioned aspects of PASC and PASC research pose unique challenges for fair subject selection, and particularly for fair inclusion, fair burden sharing, and fair opportunity. First, fair inclusion requires us to include members of a group with characteristics relevant to the disease as well as the people, or populations, most likely to have the disease [7]. Ideally, plans for subgroup analysis should be developed at the onset of the research process to ensure adequate recruitment and transparent reporting of differences and similarities between groups. An accurate conceptualization and diagnosis of the disease and tools to measure, analyze, and compare symptoms or efficacy of interventions are necessary to protect the integrity of research and ensure development of effective treatments that benefit diverse populations. The lack of definitions may lead to increased disparities in diagnosis, quality of care, as well as morbidity and mortality. Clear and accurate PASC diagnostic criteria will also be necessary to determine and design mechanisms to deliver critical social goods such as access to care, workers compensation, and disability benefits. Stigma will make the discussion of clinically relevant issues a challenge and may well adversely impact diagnosis and research recruitment.

Second, the ethical principle of fair distribution of burdens implicates that we include participants that are the best placed to bear the burdens of research (not those that have already been burdened) especially if no direct benefit is expected [7]. This principle of fair distribution of burdens seemingly conflicts with fair inclusion. For example, many individuals from underrepresented communities have been highly burdened by the COVID-19 pandemic. Stigma related to PASC will add to that burden. Nevertheless, they need to be represented for research to be generalizable to their populations. As it presently stands, with little to no benefit, if we simply “add” underrepresented populations to studies without ensuring for representation, adequate subgroup analysis and effective translation, this inclusion could constitute a form of exploitation or tokenism. In effect, researchers would be extracting data from these populations with little or no commensurate benefit to them for the risk and burden of their participation in clinical studies; the eventual treatments may well be too expensive and inaccessible.

Third, the principle of fair opportunity is linked to this notion of access to the benefits of research. Exclusion of any population from research should occur rarely and only when clearly justified for good reason (e.g., extremely high risks to individuals). Providing fair opportunity, however, is challenging because of the location of many PASC studies at high tech medical centers not accessible to all populations. Also, diagnosis by a physician or in a public health record requires access to affordable health services, something that is not available to all populations. At this time, the low likelihood of any significant benefit from participation does not offer much in the way of incentives and limits the ability of researchers to recruit participants for PASC studies. However, the burdens and benefits of PASC research will continue to evolve over time, especially with the development of interventional studies.

Lastly, the fair distribution of third-party risks may be relevant to PASC research if results seem to highlight biological or social characteristics of well-defined groups. Currently, this possibility is unknown.

## Recommendations

Since PASC is not yet well defined and potentially stigmatizing, any related research will be fraught with challenges regarding fair subject selection. Below are recommendations (also see Table 1) that may help researchers initiate conversations with community and patient stakeholders to ensure that fair inclusion, fair burden sharing, and fair opportunity are considered in research efforts. Because risks, benefits, and burdens are in large part defined by participants, collaboration as well as community engagement are critical to ensuring fair subject selection. Understanding of populations may be increased if there is a broader inclusion of researchers that represent different underrepresented groups. Indeed studies have demonstrated a homophily between identities of researchers and topics they study in the scientific literature [85,86].

**Table 1.** Recommendations for Ensuring Fair Subject Selection Across post-acute sequelea COVID-19 (PASC) Research

	PASC Characteristics	PASC study features	Ethical recommendation
Fair inclusion	PASC is not well defined Criteria that are clinically relevant are unknown, leaving researchers unsure what characteristic to include	Lack of common measurement makes researchers unsure of criteria for fair inclusion	Clarification of PASC concept, measurement criteria Real-time monitoring of studies to ensure operationalization of diversity plans
Fair burden sharing	Increased burden may be linked to diagnosis of PASC. Diagnosis may increase anxiety, discomfort, trauma or guilt Stigma may increase if study participation results in additional burden	Since studies are at large medical centers, transportation and associated costs may be a burden	More flexibility in the study protocol to reduce burden Ensure indirect benefits (e.g. compensation, social goods, access to care)
Fair opportunity	Testing/Diagnosis of COVID-19 not accessible to all Stigma of COVID-19 and PASC limits access	Studies require a COVID-19 positive test as an inclusion criteria Studies are mainly in large medical centers with PASC clinics No accountability regarding representation	Systemic considerations to create greater opportunity such as decentralization of studies beyond PASC clinics Increased support of various types of institutions to increase access and broaden participation in research Increased accountability regarding representativeness of study participants with funding to help studies meet recruitment goals

### To Address Fair Inclusion

Clarification of the concept of PASC is essential and should be based on broad evidence reflecting groups that are clinically and socially relevant. Since research on PASC is still in its infancy and often we do not know what factors may be affecting PASC, demographic, social, and biological information should be effectively gathered. Since no one study can look at all potentially relevant information in such a multisystem disorder such as PASC, diversity of studies is necessary and should include various subgroup analysis. However, diverse study should be structured in a way to ensure fair inclusion. A clear research “portfolio” is needed, that is, a research trajectory created with a series of trials or studies interrelated by common goals, metrics, outcome measures in exploratory (phases I and II), as well as confirmatory (Phase III) studies [87]. Although we cannot expect all studies to fairly include all relevant clinical and social information, we can and should expect fair inclusion within the broader portfolio.

This ideal portfolio will no doubt be even more complex with PASC given its vagueness and variety of possible endpoints for study design. However, planning this in a translational manner in which multiple sites work together to develop series of studies in a broad “portfolio” would be much more valuable and representative than any single study could be. This would require substantial restructuring of study protocols, recruitment, analysis, and knowledge translation to effectively study clinically and socially relevant populations. This may include the development of portfolios (observational, exploratory, and confirmational) that are more readily accessible to rural centers and may more directly benefit diverse populations affected by PASC. Real-time monitoring of recruitment and enrollment can ensure that some preset targets are met. These targets should be set in consultation with statisticians as well as patient and community stakeholders.

### To Address Fair Burden Sharing

In the absence of direct benefit or tangible and immediate incentives and given the unappealing potential of significant burden, researchers must be thoughtful about structuring studies that mitigate and monitor risks, minimizing burdens, and providing alternative types of benefits tailored to research participants. Efforts to reduce burden may include a focus on flexibility with scheduling to accommodate work and family obligations, as well as appropriate transportation to facilitate participation.

Alternative benefits for populations may also include compensation to offset costs related to research participation, as well as access to certain types of needed care or social goods, and financial support for long-term symptom management and reduction [88]. There is an ongoing myth that financial compensation often causes undue influence that is so grave that people – mainly those that are of lower economic status – would be unable to refuse to participate. There is no evidence that such a problem exists, and it is unjust for participants to suffer financially to take part in research. Inadequate compensation contributes to the exclusion of underrepresented groups in translational research [89].

### To Address Fair Opportunity

Study development should consider infrastructure and systemic issues, first and foremost by including individuals with PASC symptoms not being cared for at PASC clinics; these individuals may not realize – or even consider – that they have PASC. Although researchers may want to integrate studies in community clinics that are more likely to include low income and underrepresented populations, it should be acknowledged that these clinics may operate with limited funding and would require additional institutional support if research is integrated.

The COVID-19 pandemic has allowed for different modalities of research that may include telehealth to reach populations that are not in proximity to research-based hospitals. Such alternative modalities should be systematically integrated in PASC research, particularly in research that is observational, to limit burden. Reviewing how protocols could be adapted for non-hospital centers is another area of worthy future study. Although some studies may include care and technology only available in hospitals, protocol modifications may provide research opportunities to a broader demographic. If translational medicine is to improve health outcomes of populations, approaches applicable outside of hospital settings must be developed.

## Conclusion

Here we have reviewed the complexities involved in ensuring that translational research on PASC select subjects fairly. Although a protocol submitted for IRB review may have a recruitment plan that aims for diversity, this does not ensure that the study is designed and adequately resourced to assess population differences; indeed, we know that many studies do not attain their recruitment goals which also limits the ability to effectively understand differences between groups. Simply because a study secures IRB approval does not mean it will ensure downstream representative results. Furthermore, although individual IRBs can and should look at issues of diversity within research protocols [90], there is no one accountability mechanism to assess the totality of PASC research portfolios nor to provide ongoing monitoring and assessment of published work and thus no way to verify whether PASC research is structured and operationalized in a manner that adequately represents the populations it seeks to study.

The translational model requires many different studies that together – within a portfolio – result in representation, fair inclusion, fair burden sharing, and fair opportunity. And a critical first step is better tracking of recruitment data, including demographic characteristics of those informed of the opportunity to participate as well as on reasons for refusal and acceptance. By aggregating recruitment data, stakeholders can identify populations or groups that are insufficiently included (or overrepresented) and reasons in order to avoid creating and/or exacerbating long-term health disparities. Moreover, ongoing assessment and dissemination of subgroup analyses are necessary to determine which groups may require further study.

This type of accountability process within a portfolio of translational studies would require an important organizational rearrangement but would increase diversity in sampling and translatability of research to vulnerable, underrepresented, disenfranchised, or special populations. Most importantly, increasing diversity even within a restructured system should always be considered within a multifaceted fair subject selection consideration that considers fair distribution of risks, burdens, benefits, and opportunities in translational research.
